# Lactococcus garviae in Lumbar Spondylodiscitis With Spinal Epidural Abscess Causing Paraparesis: A Case Report

**DOI:** 10.7759/cureus.57827

**Published:** 2024-04-08

**Authors:** Stefan P Roch, Andreas E Zautner, Achim J Kaasch, Vanessa M Swiatek, Belal Neyazi, Klaus-Peter Stein, I. Erol Sandalcioglu, Ali Rashidi

**Affiliations:** 1 Department of Neurosurgery, Otto von Guericke University Magdeburg, Magdeburg, DEU; 2 Department of Microbiology, Otto von Guericke University Magdeburg, Magdeburg, DEU; 3 Health Campus Immunology, Infection and Inflammation (GCI), Otto von Guericke University Magdeburg, Magdeburg, DEU

**Keywords:** case report, paraparesis, lactococcus garviae, spondylodiscitis, spinal epidural abscess

## Abstract

Spinal epidural abscess (SEA) can lead to a subacute onset of neurological deficits of the extremities and is commonly accompanied by spondylodiscitis if located anterior to the dura. *Lactococcus garviae* is a fish pathogen that is occasionally found in poultry, cattle, and swine. It is a rare cause of infection in humans. Most commonly it is associated with endocarditis. Until 2019, less than 30 cases of human *Lactoccous garviae* infection have been published. To the best of our knowledge, we present the second reported case of SEA with spondylodiscitis caused by *Lactococcus garviae*. How *Lactococcus garviae* caused SEA, remains unclear in this case.

## Introduction

The occurrence of spondylodiscitis accompanied by a concurrent spinal epidural abscess (SEA) is typically rooted in bacterial infection. *Staphylococcus aureus* is the most prevalent causative agent, followed by Gram-negative bacilli, streptococci, and enterococci [1].

The incidence of SEA has been reported between 0.2 and 2.8 cases per 10,000 hospital admissions and has exhibited an upward trend. Risk factors include diabetes mellitus, immunocompromised states, trauma, chronic kidney disease, alcoholism, and intravenous substance abuse [2-4].

Patients presenting with SEA often harbor multiple risk factors. Hematogenous dissemination accounts for most infections, although in a significant proportion of cases, bacteria emanate locally from pathologies such as adjacent discitis. The identification of a clear source of infection remains elusive in many instances [5,6].

Typically, patients report acute-onset back pain and nonspecific symptoms like fever, rendering diagnosis not always straightforward. It is only with the emergence of neurological symptoms, that a diagnostic path to magnetic resonance imaging (MRI) becomes evident. Conservative management may be considered for patients with minor symptoms. This includes symptomatic therapy and most importantly adequate antibiotic treatment. Yet high failure rates often necessitate debridement surgery [7]. Conversely, neurological symptoms at onset are associated with adverse outcomes compelling the urgency of debridement surgery [4,8,9]. The aim of this case report is to raise attention towards an infection with an increasing incidence that requires quick diagnosis and adequate treatment to improve otherwise detrimental outcomes and furthermore to raise attention toward rare infectious agents like *Lactococcus garviae*, which require particular diagnostic methods for identification [10].

## Case presentation

A 79-year-old patient was referred to our university hospital's emergency department due to severe back pain and predominantly right-sided paraparesis following an unsettling outpatient lumbar MRI. Previously independent, he was now unable to walk. There were no sensory deficits or issues with bowel, urinary control, or genitoanal sensation. Laboratory studies revealed an elevated serum C-reactive protein (CRP, 119 mg/L), normal blood leukocyte counts (8.34 Gpt/L), and negative blood cultures. The patient had a history of alcoholic liver cirrhosis, esophageal variceal bleeding, recurrent ascites managed through a transjugular intrahepatic portosystemic shunt (TIPSS), and long-term rifaximin, atrial fibrillation, chronic kidney disease, insulin-dependent diabetes mellitus, and hypertension. He denied intravenous drug abuse but recalled lumbar spine injections three years ago. The patient’s occupational background involved working as a technician. During the recent two years, the patient denied any significant history of travel.

**Figure 1:**
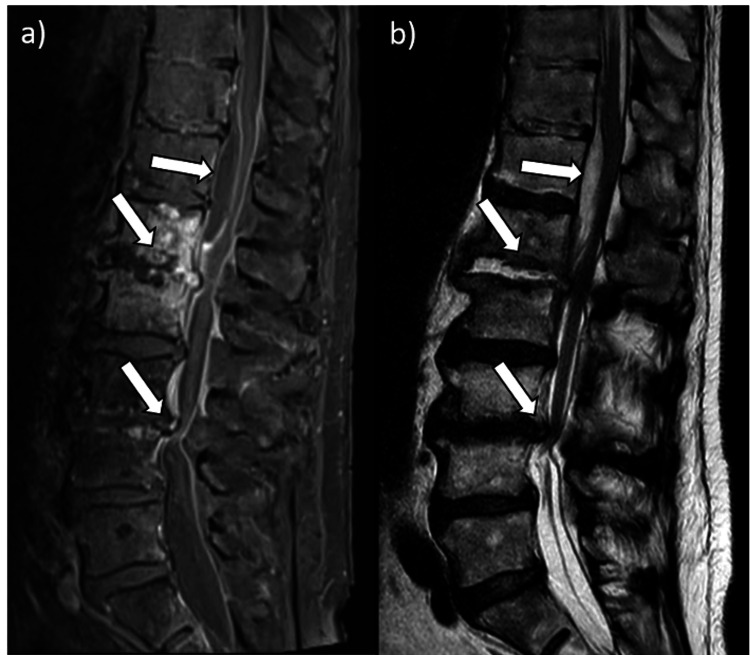
Lumbar MRI at presentation a) Sagittal T1-weighted sequence with contrast and b) Sagittal T2-weighted sequence revealing a spinal epidural abscess (SEA) at level Th12/L1, spondylodiscitis at level L1/2 and stenosis of the spinal canal at L3/4

**Figure 2:**
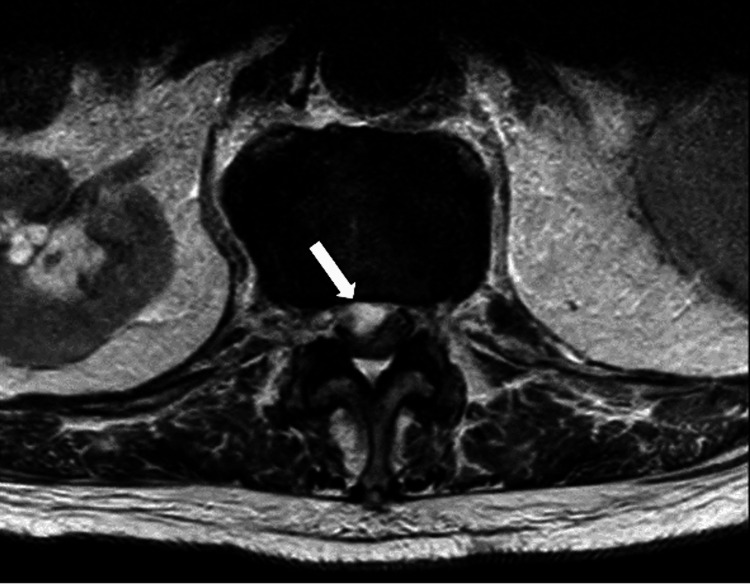
Lumbar MRI at presentation Transverse T2-weighted sequence showing high-degree spinal cord compression without cerebrospinal fluid (CSF) surrounding the spinal cord at level Th12/L1.

**Figure 3:**
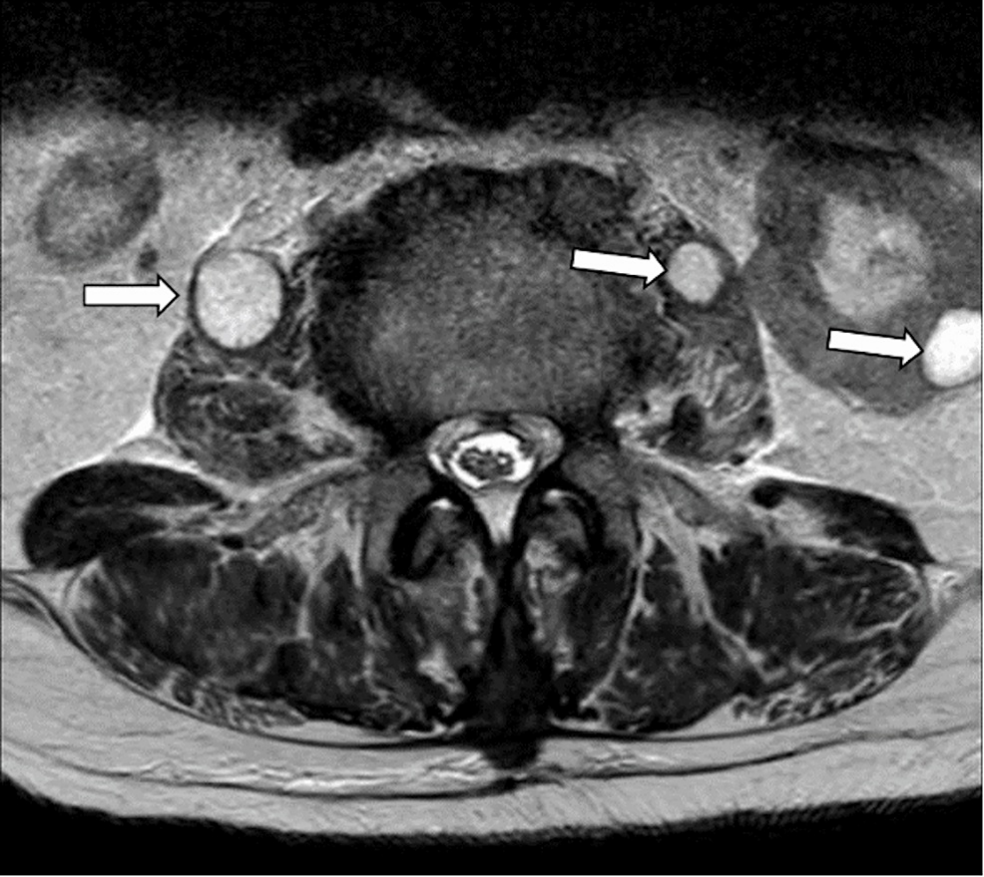
Lumbar MRI at presentation Transverse T2-weighted sequence highlighting cystic lesions in both psoas muscles and left kidney at level L2/L3.

The MRI revealed an SEA causing compression of the thoracic spinal cord at the Th12/L1 level. Additionally, spondylodiscitis at L1/L2 and discoligamentous stenosis at L3/L4 were observed (Figure 1). Axial images reveal severe spinal cord compression (Figure 2). Cystic lesions were noted in both the psoas muscles and the left kidney at the L2/L3 level (Figure 3).

We initiated antibiotic therapy with meropenem and vancomycin due to the severity of the MRI findings and neurological deficits. Urgent surgical intervention was advocated, involving debridement of the SEA and decompression of the spinal canal at L3/L4 through hemilaminectomy and undercutting to the left side. Considering the patient's anticoagulation and history of liver cirrhosis, 1,500 units of prothrombin complex concentrate were administered before surgery.

During surgery the SEA was identified, drained and microbiological samples were collected. Bacterial cultures grew *Lactococcus garviae*. Species identification was conducted using Matrix-Assisted Laser Desorption Ionization Time-of-Flight Mass Spectrometry (MALDI-TOF MS) with the MALDI Biotyper® Sirius IVD system (Bruker Daltonics GmbH & Co. KG, Bremen, Germany) with a score value of 2.31, which indicates a highly reliable identification. Post-surgery, there was a noticeable neurological improvement and reduced back pain. Due to patient incompliance, neurological rehabilitation measures such as physiotherapy faced challenges and likely led to suboptimal recovery. Serum C-reactive protein (CRP) decreased initially but later stabilized at 68 mg/L, while leukocyte counts remained normal. Antibiotic therapy with benzylpenicillin (4 million units four times daily) was administered for six weeks based on susceptibility testing results (minimum inhibitory concentration (MIC)[benzylpenicillin]=1.0 mg/L). After 10 days, antibiotic therapy was switched to ceftriaxone (2.0 g once daily) following susceptibility testing (MIC[ceftriaxone]=0.5 mg/L). Efforts to obtain further microbiological samples failed after a dry tap from attempted aspiration of the cystic psoas muscle lesion. Subsequent to a 23-day stay in the neurosurgical ward, the patient was transferred to the internal medicine department for further diagnostic workup. Transesophageal echocardiography ruled out endocarditis. Gastro- and coloscopy did not reveal local infection or malignancy. Paracentesis drained four-quadrant ascites. Ultrasound revealed reduced flow velocity in the TIPSS. The patient declined further intervention, although revision of the TIPSS was recommended.

By the fourth week of antibiotic treatment inflammatory markers stagnated. Therefore, fosfomycin was added to the antibiotic regimen. After six days in the internal medicine and infectiology ward, the patient was discharged for neurological rehabilitation, where he completed the full six-week antibiotic course of ceftriaxone and fosfomycin. Unfortunately, before the scheduled six-week follow-up MRI, the patient was readmitted due to another variceal bleed. Throughout the remaining hospitalization, repeated blood cultures yielded negative results, and there were no signs of spondylodiscitis or SEA recurrence up until 2 months after debridement surgery for SEA.

Tragically, it ended in *exitus letalis *due to the consequences of decompensated liver cirrhosis.

## Discussion

Here we present a rare case of a SEA, possibly triggered by adjacent spondylodiscitis, and caused by the uncommon bacterium *Lactococcus garviae*. Although there is a very close evolutionary relationship between Lactococcus garviae and *Lactococcus formosensis *as well as *Lactococcus petauri*, there is a minimal chance of misidentifying the microbial species by MALDI-TOF MS. In response to the patient's neurological symptoms, early debridement surgery was promptly chosen. As commonly seen in many SEA cases, no sensory nor autonomic dysfunction was present [11]. Furthermore, there was a notable spinal canal stenosis at L3/L4, concurrently addressed during the same surgical procedure. This pathology may also explain the patient’s neurological symptoms. Therefore, it is impossible to exclusively attribute his neurological symptoms to SEA with absolute certainty. 

Lumbar MRI revealed severe spinal cord compression, classified as grade 5 according to Shah et al., with an established correlation between grade 5 compression and neurological symptoms [11]. Hence, it is reasonable to infer that the patient's neurological symptoms likely resulted from the combined impact of both spinal pathologies.

Unfortunately, complete neurological recovery was not achieved, and the patient did not regain his pre-surgery level of physical activity. While he showed the ability to stand with support occasionally, the lack of compliance - refusing regular physiotherapy and assistance from nursing staff - likely hindered neurological recovery.

This challenging case presented multiple risk factors for spondylodiscitis and SEA, including diabetes mellitus, chronic kidney disease (CKD), alcoholism, an immunocompromised state due to liver cirrhosis, prior lumbar injections, and age exceeding 70 years [2,12]. Risk factors for *Lactococcus garviae* infection were also identified, such as weekly consumption of fish from a local market, pathologies of the alimentary tract like cholecystitis, and the presence of TIPSS [13]. Seeding from the TIPPS may be a possible explanation for the pathogenesis of this SEA and spondylodiscitis. The lack of TIPSS revision made it impossible to confirm this hypothesis. While the lumbar spine injections received three years ago could hypothetically serve as a possible origin, this seems less likely due to the temporal discrepancy between the infiltrations and the acute onset of the SEA. Additionally, *Lactococcus garviae *is not a known component of human skin flora [14]. 

To the best of our knowledge, documented cases of *Lactococcus garviae *causing a SEA are extremely scarce, with only one previously reported case [15]. Additionally, there is only one documented case of *Lactococcus garviae *causing spondylodiscitis without concurrent endocarditis [16]. In Table 1, we summarize cases reporting *Lactococcus garviae *as the culprit of spondylodiscitis and SEA.

**Table 1:** Review of previous documented spinal Lactococcus garviae infections without concomitant endocarditis SEA: spinal epidural abscess; CKD: chronic kidney disease; TIPSS: transjugular intrahepatic portosystemic shunt

Reference	Type of infection	Concomitant disease	Clinical presentation	Location	Potential risk factors	Treatment	Antibiotic regimen	Outcome
Amarashinge et. al. [15]	Spondylodiscitis and SEA	Non-small cell lung cancer (NSCLS)	Low-grade fever, lower back pain, no neurologic deficits	L3-L5 SEA, L5/S1 spondylodiscitis	Alcohol abuse	Surgery: hemilaminectomy L3-L5	Empiric: Vancomycin Targeted: Ceftriaxone, azithromycin six weeks total	Uncertain: death due to unrelated causes
Chan et. al. [16]	Spondylodiscitis	Chronic gastritis	Fever, lower back pain, no neurologic deficits	L3/L4	Chronic gastritis	Conservative	Ampicillin, six weeks total	Clinical resolution
Present case	Spondylodiscitis and SEA	Liver cirrhosis, spinal canal stenosis L3/L4	Lower back pain, paraparesis	Th12/L1 SEA, L1/L2 spondylodiscitis	Alcohol abuse, liver cirrhosis, TIPSS, diabetes mellitus, CKD, age > 70y, prior lumbar injections	Surgery: SEA debridement, hemilaminectomy L3/L4	Empiric: Vancomycin, meropenem Targeted: Benzylpenicillin, ceftriaxone, fosfomycin six weeks total	Uncertain: Death due to unrelated causes

The broader spectrum of *Lactococcus garviae *infections encompasses scenarios such as infective endocarditis, liver abscesses, lumbar osteomyelitis, meningitis, peritonitis, and other infections [16-18]. The rarity of this infectious agent and its diverse clinical manifestations underscore the complexity and challenges encountered in diagnosing and managing such cases. 

## Conclusions

We presented a case involving a rare infectious agent leading to a rare and serious spinal infection. Timely surgical intervention not only identified this unusual pathogen but also improved the patient's symptoms, although a full recovery wasn't achieved. Despite exhaustive investigations, the source of the infection remained unidentified. Unfortunately, follow-up imaging was not possible. However, no signs of recurrence were observed. Tragically, this case ended in *exitus letalis* due to complications unrelated to spondylodiscitis and the SEA.

This unusual case reminds us to be vigilant in everyday clinical practice and expect the unexpected. Treatment of such rare cases should always be done in close cooperation with infectious disease specialists, as we did in this case. 
